# Sestrin2 Knockdown Impairs Proliferation, Migration, Invasion, and Apoptosis in OSCC Cells via PI3K/AKT/mTOR and MAPK Pathways

**DOI:** 10.3390/cimb48010030

**Published:** 2025-12-26

**Authors:** Weijia Yang, Wangyang Wang, Zhiyuan Zhang, Zhihe Zhao, Kexin Li, Zelin Liu, Lingdan Xu, Mingxuan Shi, Yi Li, Huihui Wang

**Affiliations:** Key Laboratory of Dental Maxillofacial Reconstruction and Biological Intelligence Manufacturing, School of Stomatology, Lanzhou University, Lanzhou 730030, China; yweijia2023@lzu.edu.cn (W.Y.); wwangyang2024@lzu.edu.cn (W.W.); zhzhiyuan2024@lzu.edu.cn (Z.Z.); zhaozhh18@lzu.edu.cn (Z.Z.); 320220924150@lzu.edu.cn (K.L.); lzl960325@163.com (Z.L.); xuld16@163.com (L.X.); shimx2024@lzu.edu.cn (M.S.)

**Keywords:** sestrin2, oral squamous cell carcinoma, PI3K/AKT/mTOR pathway, MAPK pathway

## Abstract

Oral squamous cell carcinoma (OSCC) is a prevalent malignancy with a poor prognosis. Sestrin2 (Sesn2), a stress-inducible protein, has been implicated in various cancers, but its precise role and mechanism in OSCC remain unclear. This study investigated the molecular mechanisms of Sesn2 in OSCC. Sesn2 expression was analyzed using data from TCGA and immunohistochemical results from the HPA. Functional assays, including CCK-8, flow cytometry for cell cycle, wound healing, and Transwell assays, were performed following *Sesn2* knockdown with siRNA in OSCC cell lines (CAL-27 and SAS). Underlying mechanisms were investigated by Western blotting and ELISA for MMP-2 and MMP-9 levels. Sesn2 was significantly upregulated in OSCC tissues compared to normal controls. Its knockdown markedly suppressed cell proliferation, induced G1 phase cell cycle arrest, and impaired migratory and invasive capabilities. This reduction in invasion was further confirmed by decreased levels of MMP-2 and MMP-9 upon *Sesn2* knockdown. Furthermore, *Sesn2* silencing induced apoptosis via Caspase-3 activation with divergent BAX/BCL-2 modulation; SAS cells exhibited elevated BAX and reduced BCL-2, whereas these proteins remained unchanged in CAL-27 cells. Mechanistically, we found that Sesn2 depletion downregulated the PI3K/AKT/mTOR pathway and reduced the phosphorylation of AKT and p38 MAPK. Our findings demonstrate that Sesn2 functions as an oncogene in OSCC, promoting tumor progression by modulating the PI3K/AKT/mTOR and MAPK signaling pathways, suggesting its potential as a therapeutic target for OSCC.

## 1. Introduction

Approximately 600,000 people worldwide are diagnosed annually with head and neck squamous cell carcinoma (HNSCC), accounting for 5.5% of all cancers, and it has a 5-year survival rate of ~50% [1,2]. Oral cancer is the most common cancer of the head and neck. Forming 90% of all cases of oral cancer, oral squamous cell carcinoma (OSCC) is the most prevalent type [3]. With high mortality and low cure rates, OSCC is a major public health problem with enormous personal and socio-economic impacts [4]. At present, surgery combined with chemotherapy, radiotherapy, biotherapy, and other adjuvant therapies is the primary clinical method for the management of OSCC [5,6]. Due to distant metastases, regional recurrence, and resistance to radiotherapy and chemotherapy, the survival rates of OSCC have not improved despite advances in the diagnosis and treatment of this disease [7,8]. Thus, exploring the etiopathogenesis of OSCC and discovering novel therapeutic targets remains an important approach in the long-term goal of improving the therapeutic outcomes of patients with OSCC.

OSCC is characterized by extensive molecular heterogeneity, encompassing aberrant activation of multiple signaling cascades and widespread dysregulation of gene expression. For instance, the Wnt/β-catenin pathway is instrumental in OSCC metastasis, with miR-223 modulating this axis to facilitate malignant progression [9]. Hypoxia further amplifies the invasive, migratory, and angiogenic capacity of OSCC cells, in which Suprabasin plays a pivotal role [10]. The PI3K/AKT/mTOR pathway is also central to OSCC pathogenesis; its hyperactivation sustains tumor-cell proliferation, survival, metabolic reprogramming, and motility. Panx3 is frequently downregulated in OSCC, and its re-expression can trigger ferroptosis via the AKT/mTOR network [11]. Parishin A exerts tumor-suppressive effects in OSCC through concurrent inhibition of the AKT/mTOR cascade [12]. Additionally, interleukin-8 (IL-8) promotes metastasis and chemoresistance by activating the Akt/Erk–NF-κB axis [13]. These intertwined molecular events collectively drive OSCC initiation, progression, and therapeutic refractoriness. Sestrin2 (Sesn2) is an important antioxidant protein with a molecular weight of 54.5 kDa. The gene is located on chromosome 1, at Cytoband p35.3, and encodes a 480 amino acid protein [14]. Structurally, Sesn2 consists of two globular-like α-helical subdomains, the N-terminal domain, and the C-terminal domain, which are connected by a helix-loop-helix motif. The N-terminal domain possesses antioxidant activity, while the C-terminal domain contains a binding site for leucine [15]. Sens2 functions as a positive regulator of autophagy to activate the antioxidant system and maintain cell homeostasis [16,17]. It has been shown that when the liver is stimulated by acute lipogenesis, Sesn2 can inhibit autophagic degradation of Keap1 through binding to Keap1 and p62/SQSTM, which leads to activation of the nuclear factor Nrf2, thus activating the antioxidant system [18]. There are also reports indicating that Sesn2 can reduce oxidative stress injury by activating the AMPK pathway, protecting mitochondrial function, and limiting JNK-NFκB signal transduction mediated by reactive oxygen species in ischemic heart disease [19,20]. In addition, Sesn2 acts as a stress-induced metabolic regulator that could regulate cell growth and metabolism, contributing to the prevention of multiple diseases. Specifically, in physiological damage and aging, as well as in various metabolic disorders (such as diabetes, cardiac hypertrophy, and atherosclerosis), Sesn2 can activate the AMPK/mTOR signaling pathway to maintain metabolic homeostasis and reduce cell proliferation [21]. As a key orchestrator of cellular stress responses, Sesn2 modulates AKT/mTOR signaling and dictates cell fate decisions.

The function of Sesn2 exhibits pronounced heterogeneity across different cancer types, and this dual role constitutes a central challenge in its translational investigation [22]. Sesn2 acts as a cancer biomarker and potential therapeutic target, playing a critical role in cancer occurrence and progression [23,24]. Sesn2 has been shown to function as a tumor suppressor gene in bladder cancer and prostate cancer [22,25,26]. However, several more recent studies revealed that higher levels of Sesn2 expression are found in melanoma and squamous cell carcinoma [27] compared to noncancerous tissues, and the increased expression was associated with a poorer prognosis [28]. Additionally, another study showed that Sesn2 promotes AKT activation in melanoma and skin squamous carcinoma while exerting oncogenic effects by promoting cell viability under UVB stress and chemotherapy [27]. AKT signaling critically regulates cell proliferation, survival, metabolism, and migration; its sustained activation is frequently observed in diverse malignancies and predicts adverse clinical outcomes. In OSCC, pharmacologic or genetic inhibition of the PI3K/AKT/mTOR cascade effectively suppresses tumor-cell proliferation, invasion, and metastatic dissemination [11,12,13]. These attributes render Sesn2 a compelling candidate for in-depth investigation in OSCC, with the potential to clarify its precise impact on tumor progression and to identify novel therapeutic targets. In short, Sesn2 function differs in different types of cancer [29]. Nevertheless, the expression of Sesn2 and its specific role in OSCC remains unknown.

This study investigated the function and mechanism of Sesn2 in OSCC in vitro. Sesn2 expression and its clinicopathological relevance were analyzed using publicly available datasets. We knocked down *Sesn2* in two OSCC cell lines (CAL-27 and SAS) and assessed its effects on proliferation, migration, invasion, and apoptosis. Western blotting was performed to evaluate Sesn2-mediated modulation of the PI3K/AKT/mTOR pathway. Our findings elucidate the role of Sestrin2 in OSCC and support its potential as a therapeutic target.

## 2. Materials and Methods

### 2.1. Bioinformatics Analysis

The clinical information and RNA-seq data in OSCC were obtained from The Cancer Genome Atlas (TCGA) [30]. The samples from oral cancers (alveolar ridge, base of tongue, buccal mucosa, floor of mouth, hard palate, oral cavity, and oral tongue) were included, and the samples from non-oral cancers (hypopharynx, larynx, lip, oropharynx, and tonsil) were excluded. RNA-seq data was converted into the TPM format, and log2 conversion was performed. The survival analysis and the association between Sesn2 expression levels were assessed using the R package (ggplot2, version 3.3.3). Statistical differences in the paired and the unpaired samples were assessed using Student’s *t*-test. Statistical differences in survival analysis were assessed using the log-rank test. The images of Sesn2 immunohistochemical staining were obtained from the Human Protein Atlas (HPA) database https://www.proteinatlas.org (accessed on 12 December 2025). The immunohistochemical samples of normal oral mucosa tissue with patient ID 2547 and HNSCC with sample ID 1743 were extracted, and the HPA018191 antibody was used for staining. The clinical samples and patient information utilized in this study were obtained from publicly accessible databases, thereby exempting the need for review by the Ethics Committee. Immunohistochemical pictures of HNSCC showed lymph node metastasis of tongue squamous cell carcinoma.

### 2.2. Cell Cultures

The human tongue squamous carcinoma cell lines, CAL-27 and SAS, were purchased from Cellcook Biotech (CC0701 and CC0706, Guangzhou, China). The cells were cultured in DMEM (L110KJ, BasalMedia, Shanghai, China) supplemented with 10% FBS (ABW, Shanghai, China) and 1% penicillin-streptomycin solution (S110JV, BasalMedia, Shanghai, China), and maintained in a humidified incubator supplied with 5% CO_2_ at 37 °C. All cells used in the experiment were negative for mycoplasma, and no more than 20 passages were used.

### 2.3. Cell Transfection

For transfection, CAL-27 and SAS cells were seeded in T25 flasks at a density of 5 × 10^5^ cells per flask and cultured overnight. Transfections were performed using Lipofectamine^®^ 2000 and Opti-MEM medium according to the manufacturer’s protocol (11668030 and 31985070, Thermo Fisher Scientific, Waltham, MA, USA). Small interfering RNA (siRNA) was obtained from GenePharma (Shanghai, China) (sequences in Table 1). After 4–6 h of transfection, the medium was replaced with fresh complete medium. To validate the knockdown efficiency, total protein was extracted 48 h post-transfection, and the expression level of Sesn2 was assessed by Western blotting. Subsequent functional experiments were conducted within 24 to 72 h after transfection. The siRNA that demonstrated significant knockdown efficiency was selected for all functional assays.

### 2.4. Cell Counting and Cell Proliferation Assay

After 24 h of transfection of CAL-27 and SAS cells, each 96-well plate was seeded with 3000 cells. Cells were cultured for 24, 48, or 72 h, after which, 10 μL CCK-8 solution (K1018, Apexbio, Houston, TX, USA) was added. Following 2 h of incubation, a microplate reader Infinite^®^ M200 Pro (Tecan, Männedorf, Switzerland) was used to measure the absorbance at 450 nm. Experiments were performed with six technical replicates per condition and independently repeated at least three times.

### 2.5. Cell Cycle Assay

Cells were collected, resuspended in PBS, and fixed overnight with 70% ethanol at 4 °C. Subsequently, propidium iodide (PI) staining (C1052, Beyotime, Shanghai, China) was performed for 30 min in the dark in a water bath with a temperature of 37 °C, followed by flow cytometry analysis (CytoFLEX, Beckman Coulter, Indianapolis, IN, USA). A strict gating strategy was applied for data acquisition. First, a single-cell population (Gate P1) was gated on the forward scatter area (FSC-A) vs. side scatter area (SSC-A) plot to exclude cell debris and clumps; then, a viable single-cell subset (Gate P2) was further defined on the FSC-H vs. FSC-A plot to eliminate adherent cells. Only the signals of cells within Gate P2 were exported to the PI fluorescence intensity histogram (PE-A channel) for cell cycle analysis.

For distinguishing dual-peak signals in the histogram, the peak with lower fluorescence intensity was identified as the G0/G1 phase (diploid DNA content), while the peak at approximately twice the fluorescence intensity was designated as the G2/M phase (tetraploid DNA content); the plateau region between the two peaks represented the S phase (cells undergoing DNA synthesis). A total of 10,000 valid events were collected from each sample to ensure statistical reliability. Cell cycle phase distribution was analyzed using FlowJo software (V10.8, BD Biosciences, San Jose, CA, USA). The percentage of cells in each phase was calculated using the following formula. The software automatically generated the proportions of cells in G0/G1, S, and G2/M phases. All experiments were performed in triplicate, and the mean values were used for subsequent statistical analysis.

### 2.6. Cell Migration and Invasion

A total of 1 × 10^6^ cells were seeded per well in a six-well plate and cultured until 100% confluence was reached. A straight wound was then created in the monolayer using a sterile pipette tip. After washing to remove detached cells, the cells were incubated in serum-free medium. Photographs of the wound area were taken immediately after scratching (0 h) and at 24 h and 48 h of incubation to monitor migration.

Next, Transwell assays were used to further assess the migratory and invasive ability of cells. Cells in the logarithmic growth phase were plated in the upper chamber of a Transwell insert (100,000 cells per well) (8.0 μm pore size, Corning, Corning, NY, USA.) in 200 μL media without serum. In the lower chamber of the inserts, 700 μL supplemented media was added. After 24 h of incubation (37 °C, 5% CO_2_), cells were fixed in 4% paraformaldehyde at room temperature for 30 min and stained using Giemsa (G1015, Solarbio, Beijing, China) for 15 min at room temperature. The inner of the upper chamber cells was wiped off with a wet cotton swab, and the cells that had migrated were observed and counted using a light microscope (Magnification of 20 × 10). For the invasion experiments, 60 μL 1 mg/mL ECM Gel (E1270, Merck, Darmstadt, Hessen, Germany) was added to the upper chamber of the Transwell inserts first (8.0 μm pore size, Corning, Corning, NY, USA), and 200 μL serum-free cell suspensions containing 100,000 cells were added after 2–3 h. The rest of the procedure was the same as that for the migration experiments, but the invasion experience was incubated for 36 h. Finally, the image was quantified by counting five random fields under a microscope (Olympus, Tokyo, Japan) using ImageJ (v1.8.0.322; National Institutes of Health, Germany), with triplicate wells per condition, which were randomly selected. Each biological experiment was independently repeated three times.

### 2.7. Apoptosis Detection

A total of 2 × 10^5^ CAL-27 and SAS cells transfected with small interfering RNA reagents were plated per well in a 24-well plate. Hoechst 33342 staining solution (C0031, Solarbio, Beijing, China) was left at room temperature for 5–10 min, washed with PBS 3 times, and the process was repeated three times; cells were subsequently observed using a fluorescence microscope. After 48 h of transfection with small interfering RNA reagents, CAL-27 and SAS cells were trypsinized, centrifuged (200 g, 5 min, RT), and washed twice with 1 mL ice-cold PBS. Cell aliquots (2 × 10^5^ cells) were resuspended in 195 µL Annexin V-FITC binding buffer, followed by sequential addition of 5 µL Annexin V-FITC and 10 µL PI (C1062S, Beyotime, Shanghai, China) with gentle mixing. After 10–20 min of incubation in darkness (20–25 °C) with intermittent resuspension (2–3 times), samples were placed on ice and analyzed within 1 h using CytExpert (Beckman Coulter, Indianapolis, IN, USA). Triplicate independent experiments were performed for statistical analysis.

### 2.8. Western Blotting

Total protein was extracted from cells using high-efficiency RIPA lysis buffer supplemented with 1% PMSF and 1% protein phosphatase inhibitor cocktail (Solarbio, Beijing, China). Protein concentration was determined using a BCA Assay Kit (CW0014S, CWBIO, Taizhou, China). Equal amounts of protein (40 μg per lane) were separated by 10% SDS-PAGE and subsequently transferred onto PVDF membranes (Cytiva, Marlborough, MA, USA). The membranes were blocked with 5% skim milk (BD Biosciences, San Jose, CA, USA) in TBST (Tris-buffered saline with 0.05% Tween-20) for 1.5 h at room temperature. Thereafter, membranes were incubated overnight at 4 °C with the following primary antibodies (all from ProteinTech, Wuhan, China) diluted in blocking buffer: anti-GAPDH (1:7000), anti-Sesn2 (1:2000), anti-PI3K (1:1000), anti-AKT (1:5000), anti-phospho-AKT (Ser473, 1:3000), anti-p38 MAPK (1:5000), anti-phospho-p38 MAPK (Thr180/Tyr182, 1:3000), anti-BCL-2 (1:3000), anti-BAX (1:3000), and anti-Caspase-3 (1:1000). After three washes with TBST, membranes were incubated with appropriate horseradish peroxidase-conjugated secondary antibodies for 2 h at room temperature. Protein bands were visualized using an enhanced chemiluminescence substrate (Yeasen Biotechnology, Shanghai, China), and the signal intensities were quantified using ImageJ.

### 2.9. ELISA

Total MMP2 and MMP9 levels in cell culture supernatants, collected 48 h post-transfection, were quantified using ELISA kits (KE00077 and KE00456, ProteinTech, Wuhan, China) according to the manufacturer’s protocols. Absorbance was measured at 450 nm, and analyte concentrations were determined directly from the standard curve. Each experiment was performed in triplicate.

### 2.10. Caspase-3 Activity Assay

Caspase-3 activity was determined using the Caspase-3 Activity Assay Kit. Briefly, cells were lysed in buffer, and homogenates were harvested by centrifugation at 12,000× *g*. Protein concentrations were measured using the Bradford Protein Assay Kit (C1115 and P0006, Beyotime, Shanghai, China). Subsequently, cell lysates were incubated with Ac-DEVD-pNA (2 mmol/L) at 37 °C for 24 h. Caspase-3 activity was then quantified by measuring absorbance at 405 nm on a microplate reader.

### 2.11. Statistical Analysis

Bioinformatics analyses, including differential expression and survival analysis from public databases, were conducted with R software (version 3.6.3). For the analysis of data obtained from in vitro experiments, GraphPad Prism (version 8.3.0) was used. In Bioinformatics analysis, Statistical differences in the paired and the unpaired samples were assessed using Student’s *t*-test. Statistical differences in survival analysis were assessed using the log-rank test. In in vitro experiments, statistical differences between the two groups were assessed using Student’s *t*-test. Two-way ANOVA (group × time) was assessed for multiple time points. For post hoc pairwise comparisons following ANOVA, Tukey’s Honestly Significant Difference (HSD) test was selected in the case of homogeneous variances, while Dunnett’s T3 test was adopted for the scenario of heterogeneous variances. Data normality was assessed via the Shapiro–Wilk test. Both post hoc tests include built-in corrections for multiple comparisons, eliminating the need for additional α-level adjustments. Data from at least three independent experiments are presented as the mean ± standard deviation (SD). A *p*-value of less than 0.05 was considered statistically significant.

## 3. Results

### 3.1. Sesn2 Expression Levels in OSCC

**Figure 1:**
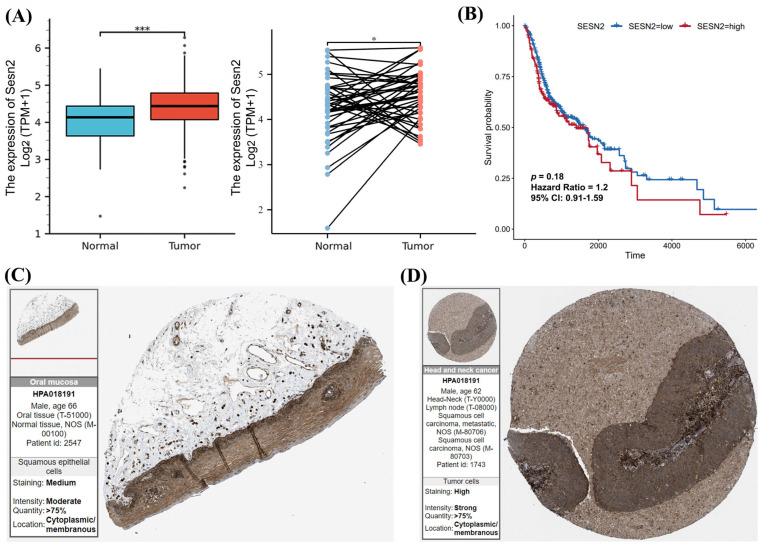
The Sesn2 expression in OSCC. (**A**) The differential analysis of Sesn2 expression in tumor and normal cells by unpaired and paired samples. (*, *p* < 0.05; ***, *p* < 0.001). (**B**) Kaplan–Meier survival curves of OSCC patients stratified by Sesn2 expression level. (**C**) The immunohistochemical staining of Sesn2 in normal oral mucosa tissues. (**D**) The immunohistochemical staining of Sesn2 in OSCC tissues.

Based on TCGA data analysis, after excluding data without clinical information, the expression of *Sesn2* mRNA in normal tissues (*n* = 32) was significantly lower than in OSCC tissues (*n* = 329) (*p* < 0.001, Figure 1A). Subsequently, according to the screened 32 paired samples, expression was lower in the healthy tissues compared to the cancerous tissues (*p* < 0.01, Figure 1A). Survival analysis showed that the survival probability of the low Sesn2 expression group tended to be better than that of the high Sesn2 expression group, but there was no significant difference (Figure 1B). We next utilized the HPA database to examine Sesn2 protein expression, comparing normal oral mucosa tissue to OSCC tissue. Immunohistochemical pictures of HNSCC showed lymph node metastasis of tongue squamous cell carcinoma. Immunohistochemical staining showed that the expression level of Sesn2 might be higher in OSCC tissues (Figure 1C,D).

### 3.2. CAL-27 and SAS Proliferation Is Reduced Following Sesn2 Knockdown

**Figure 2:**
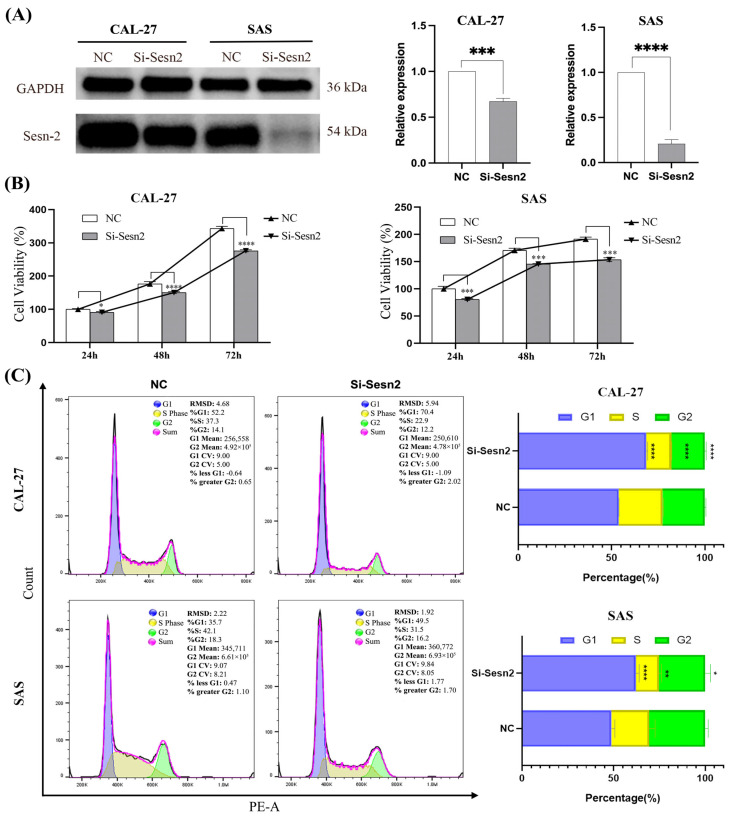
Inhibited proliferation of CAL-27 and SAS cells by Sesn2 knockdown. (**A**) Protein expression levels after Sesn2 knockdown. (**B**) The effects of Sesn2 knockdown at different times (24 h, 48 h, 72 h) on cell counting. (**C**) The effects of Sesn2 knockdown on the cell cycle. The results were based on at least three independent experiments. Student’s *t*-test and two-way ANOVA were applied for statistical analysis. (mean ± SD, *, *p* < 0.05; **, *p* < 0.01; ***, *p* < 0.001; ****, *p* < 0.0001; vs. NC; *n* = 3).

Western blot analysis confirmed that transfection with si-Sesn2 markedly reduced Sesn2 protein expression in both Cal-27 and SAS cell lines. (Figure 2A). The results of the CCK-8 assay showed that proliferation was lower in the Si-Sesn2 group than in the NC group, and this effect was time-dependent (Figure 2B). Flow cytometry of the cell cycle further revealed that the proportion of cells in the G0/G1 phase increased significantly in the Si-Sesn2 group (Figure 2C).

### 3.3. Migration and Invasion in CAL-27 and SAS Are Reduced Following Sesn2 Knockdown

**Figure 3:**
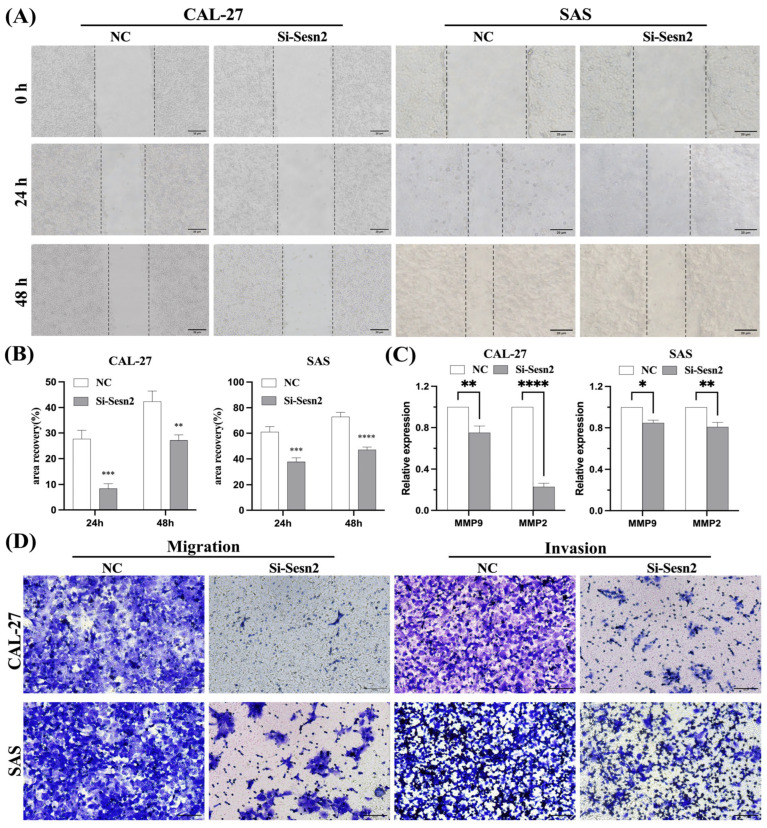
*Sesn2* knockdown inhibited migration and invasion in CAL-27 and SAS cells. (**A**) Representative images of scratch wounds at 0 and 24 h to reflect cell migration ability. (Bar = 20 μm). (**B**) Quantification of migration rates in CAL-27 and SAS cells. (**C**) MMP-2 and MMP-9 expression levels. (**D**) Transwell assays and cell invasion experiments evaluated cell migration and invasion. (Bar = 100 μm). The results were based on at least three independent experiments. Student’s *t*-test was applied for statistical analysis. (mean ± SD, *, *p* < 0.05; **, *p* < 0.01; ***, *p*< 0.001; ****, *p* < 0.0001; vs. NC; *n* = 3).

To assess the impact of *Sesn2* silencing on CAL-27 and SAS cell motility, Representative wound healing images were used to assess cell migration. Si-Sesn2 significantly impaired the migration of both cell lines (Figure 3A,B). ELISA confirmed that MMP-2 and MMP-9 expression was markedly reduced following *Sesn2* knockdown (Figure 3C). Si-Sesn2 suppressed their migration and invasion capacities (Figure 3D).

### 3.4. Apoptosis of CAL-27 and SAS Cells Is Increased Following Sesn2 Knockdown

**Figure 4:**
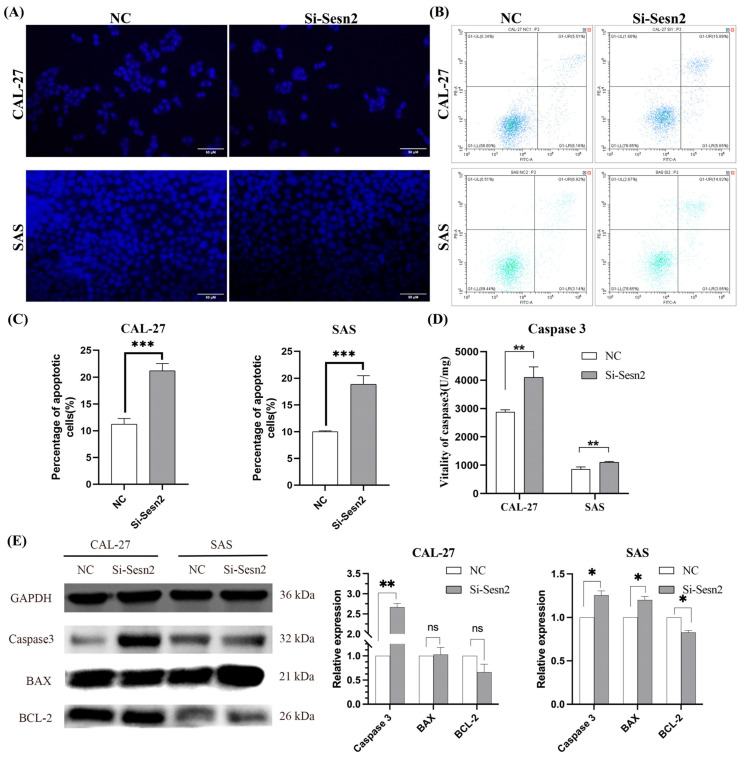
*Sesn2* knockdown induced apoptosis in CAL-27 and SAS cells. (**A**) Hoechst 333342 staining observes changes in apoptosis morphologically. (Bar = 50 μm). (**B**) Annexin V-FITC/PI flow cytometry plots for CAL-27 and SAS cells. (**C**) Quantification of apoptosis rates in CAL-27 and SAS cells. (**D**) Caspase-3 activity in CAL-27 and SAS cells following *Sesn2* knockdown. (**E**) Total Caspase-3, BAX, and BCL-2 protein expression detection. The results were based on at least three independent experiments. Student’s *t*-test was applied for statistical analysis. (mean ± SD, *, *p* < 0.05; **, *p* < 0.01; ***, *p* < 0.001; ns: no significance vs. NC; *n* = 3).

*Sesn2* knockdown induced apoptosis, as evidenced by increased apoptotic bodies and concomitantly reduced cell number on Hoechst 33342 staining (Figure 4A). Annexin V-FITC/PI flow cytometry demonstrated a significantly higher apoptotic rate in the si-Sesn2 group compared with the NC group (Figure 4B,C). Caspase-3 activity assays revealed elevated active Caspase-3 levels following *Sesn2* silencing (Figure 4D). Western blot analysis further confirmed upregulated total Caspase-3 expression in both CAL-27 and SAS cells. Notably, BAX and BCL-2 levels remained unchanged in CAL-27 cells, whereas SAS cells exhibited elevated BAX and reduced BCL-2 expression (Figure 4E).

### 3.5. Knockdown of Sesn2 Alters the Expression Levels of Key Proteins Involved in the PI3K/AKT/mTOR and MAPK Pathway

**Figure 5:**
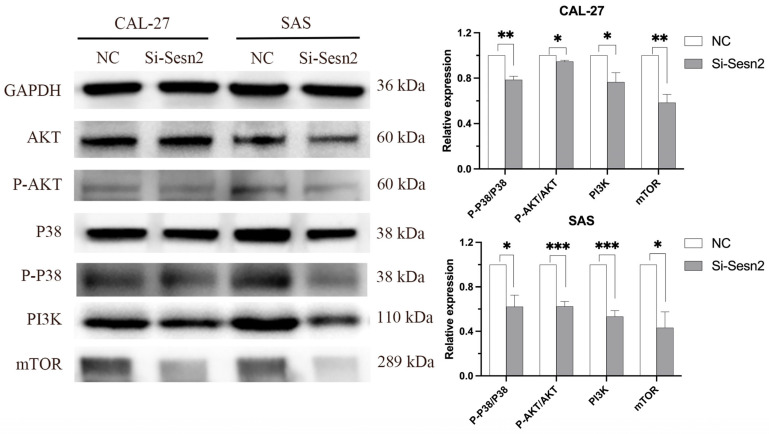
*Sesn2* changes the expression level of key proteins in the mTOR signal pathway in CAL-27 and SAS cells through Western blotting. The results were based on at least three independent experiments. Student’s *t*-test was applied for statistical analysis. (mean ± SD, *, *p* < 0.05; **, *p* < 0.01; ***, *p* < 0.001; vs. NC; *n* = 3).

To investigate the effects of *Sesn2* knockdown on the proliferation, migration, invasion, and apoptosis of OSCC cell lines, Western blotting was used to explore the potential mechanisms regulated by *Sesn2*. The results showed that *Sesn2* altered the expression levels of key proteins in the mTOR pathway and reduced the expression of mTOR, PI3K, P-AKT, and P-P38 (Figure 5).

## 4. Discussion

The most common malignancy of the head and neck is OSCC, which is difficult to treat due to its complex etiology. Moreover, due to difficulties in speech, cosmetic appearance, and eating habits, a patient’s quality of life can be notably affected [31]. To date, there are no effective means to solve this problem. In the present study, Sesn2 expression data in TCGA and immunohistochemical staining data from the HPA database were analyzed, which showed that Sesn2 expression was upregulated in oral cancer specimens compared with normal control specimens. Survival analysis suggested that Sesn2 might be a risk factor, and the curve of the low expression group was basically higher than that of the high expression group, but the results showed no statistical difference. This observation may be ascribed to the inadequate sample size inherent in the database. This suggests that Sesn2 might have some auxiliary significance, and it may be a potential target worthy of further study in OSCC. In the majority of studies, Sesn2 primarily plays an inhibitory role in cancer, but it has also been reported that Sesn2 promotes the development of cancer [32], such as pancreatic cancer [33], liver cancer [34], and melanoma [35]. The present study demonstrated that *Sesn2* knockdown significantly suppressed the proliferation of CAL-27 and SAS cells, as evidenced by CCK-8 assays. This anti-proliferative effect was further determined to be attributable to a cell cycle arrest at the G0/G1 phase. This finding prompts an exploration into the underlying molecular mechanisms connecting Sesn2 to cell cycle progression.

The effect of Sesn2 on tumor migration and invasion was also studied. In the wound healing and Transwell assays, the migratory rate and the number of CAL-27 and SAS cells that had migrated following the knockdown of *Sesn2* decreased significantly, indicating that the migratory and invasive ability of CAL-27 and SAS cells was reduced following *Sesn2* knockdown. MMPs are zinc-dependent endocrine proteases that cleave extracellular matrix proteins and play a key role in tumor cell migration, diffusion, tissue invasion, and metastasis [36,37,38]. The process of cell migration and invasion is highly dependent on the function of MMPs. To further verify the role of MMPs in these processes, the expression levels of MMP2 and MMP9 were determined, and the results showed that their expression at the protein level had decreased. Based on the results of the present study, combined with the known roles of MMPs, it was hypothesized that the pro-oncogenic role of *Sesn2* in OSCC involves the upregulation of MMP2 and MMP9 to facilitate cell migration and invasion. Apoptosis plays an important role in the occurrence, development, and acquisition of drug resistance of tumors, and inducing apoptosis is an important strategy in tumor therapeutics [39,40]. The system regulating apoptosis in cells is very complex, in which Caspases play a key role by destroying a variety of anti-apoptotic factors [41]. The results showed that apoptotic bodies, nuclei fragmentation, and Caspase-3 protein level all increased following *Sesn2* knockdown, indicating an increase in apoptosis of CAL-27 and SAS cells. Caspase-3 is the executor of apoptosis, and its activation can induce apoptosis [42,43]. In CAL-27 cells, the unchanged BAX/BCL-2 ratio indicates minimal mitochondrial involvement, yet Caspase-3 activation was observed, suggesting that *Sesn2* knockdown may promote apoptosis through the death receptor pathway. As a key regulator of growth and adaptation to environmental stress signal transduction, the mTOR complex 1 plays a crucial role in receiving growth factors, nutrition, energy, and other signals, and it is often activated in human cancer [44]. In the present study, following the knockdown of Sesn2 expression, the expression of mTOR was also reduced. Furthermore, cells became arrested in the G1 phase, and apoptosis was triggered following mTOR signal pathway inhibition. This is consistent with a previous study, in pancreatic cancer, where Sesn2 can increase glycolysis and promote the proliferation of pancreatic cancer cells, and both of these pro-tumorigenic effects were reversed by mTOR inhibitors [33]. However, in colon cancer and endometrial cancer cell lines, the increased expression of Sesn2 can inhibit cancer by inhibiting mTOR [45,46]. The differential expression of Sesn2 in different tumor tissues and cell lines may be associated with the different activation mechanisms, and its differing effects may be associated with the activation states of several downstream signaling pathways regulated by it.

**Figure 6:**
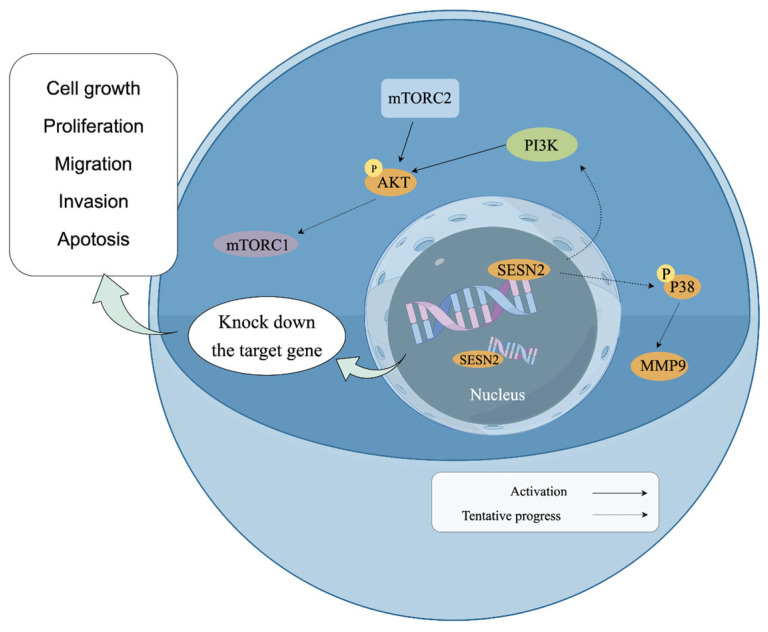
Schematic model diagram of Sesn2 in the PI3K/AKT/mTOR and MAPK path (By Figdraw).

To explore the molecular mechanism underlying the effects of Sesn2 on the proliferation, migration, invasion, and apoptosis of CAL-27 and SAS cells, the key proteins in the mTOR pathway were further studied. It is well established that the upstream effectors of mTOR are classical PI3K/AKT and Ras/Raf/MEK/ERK (MAPK) molecules [47,48]. The results of the present study showed that the expression of PI3K and phosphorylated AKT decreased. Inhibition of PI3K/AKT can inhibit cell proliferation and metabolism and promote apoptosis by activating downstream apoptotic proteins. A previous study showed that Ras/Raf/MEK/ERK promoted MMP2 and MMP9 transcription by activating transcription factor AP-1 [49,50]. In the present study, the expression of MMP2 and MMP9 decreased, and the levels of P38 decreased. This suggested that *Sesn2* knockdown may have suppressed P38 activation, which in turn likely inhibited the transcription factors responsible for MMP2 and MMP9 expression, thereby reducing cell migration and invasion [51]. However, these results contradict those of another previous study in which P38 expression was decreased and apoptosis increased [52], though this may be attributed to the fact that P38 primarily plays a role in the migration of CAL-27 and SAS cells in the present study, and apoptosis was regulated by mTOR and PI3K/AKT instead (Figure 6).

In conclusion, this study demonstrates that *Sesn2* knockdown suppresses proliferation, migration, and invasion while inducing apoptosis in oral tongue squamous cell carcinoma, potentially through modulation of the PI3K/AKT/mTOR and MAPK signaling pathways. These results implicate *Sesn2* as a promising therapeutic target for OSCC. Nevertheless, several limitations constrain our findings. First, our study is limited to two cell lines from the same anatomical region, reducing generalizability to other head and neck cancer subtypes. Second, the absence of vivo validation precludes definitive assessment of therapeutic relevance. Third, the lack of rescue or overexpression studies hinders mechanistic interpretation. Future investigations incorporating in vivo models, complementary gain-of-function approaches, and comprehensive pathway mapping will be essential to fully validate the clinical potential of Sesn2-targeted therapy.

## Figures and Tables

**Table 1:** Target sequence for knockdown of the human *Sesn2* gene.

Gene Name	Sequence (5′-3′)	
	*Sesn2*	Anti-*Sesn2*
*Si-Sesn2*	GGAACCUCAAGGUCUAUAUTT	AUAUAGACCUUGAGGUUCCTT
Negative control	UUCUCCGAACGUGUCACGUTT	ACGUGACACGUUCGGAGAATT

## Data Availability

The raw data supporting the conclusions of this article will be made available by the authors on request.

## Abbreviations

The following abbreviations are used in this manuscript:

AKT	Protein kinase B
AMPK	5′-adenosine monophosphate-activated protein kinase
AP-1	Activator protein-1
BAX	BCL-2-associated X protein
BCL-2	B-cell leukemia/lymphoma-2
ERK	Extracellular signal-regulated kinase
OSCC	Oral squamous cell carcinoma
HNSCCs	Head and neck squamous cell carcinomas
JNK	c-Jun N-terminal kinase
MAPK	Mitogen-activated protein kinase
MEK	Mitogen-activated protein kinase kinase
mTOR	Mammalian target of rapamycin
MMPs	Matrix metalloproteinases
MMP2	Matrix metalloproteinase-2
MMP9	Matrix metalloproteinase-9
NF-κB	Nuclear Factor-κB
Nfr2	Nuclear factor (erythroid-derived 2)-like 2
p-AKT	Phosphorylated kinase B
PI3K	Phosphoinositide 3-kinase
Raf	Rapidly accelerated fibrosarcoma
Ras	Rat sarcoma virus
Sesn2	Sestrin2
